# A Pilot Study on the Age-Dependent, Biomechanical Properties of Longitudinal Ligaments in the Human Cervical Spine

**DOI:** 10.3390/bioengineering12010061

**Published:** 2025-01-13

**Authors:** Narendra Singh, Ana Trajkovski, Jovan Trajkovski, Robert Kunc, Jose Felix Rodriguez Matas

**Affiliations:** 1 Chair of Modelling in Engineering Sciences and Medicine, Faculty of Mechanical Engineering, University of Ljubljana, Aškerčeva c. 6, 1000 Ljubljana, Slovenia; ana.trajkovski@fs.uni-lj.si (A.T.); jovan.trajkovski@fs.uni-lj.si (J.T.); robert.kunc@fs.uni-lj.si (R.K.); 2 Department of Chemistry, Materials and Chemical Engineering “Giulio Natta”, Politecnico di Milano, Piazza Leonardo da Vinci, 32, 20133 Milano, Italy; josefelix.rodriguezmatas@polimi.it

**Keywords:** human cervical spine, ligament, tension test, age-related changes, biomechanics, mechanobiology, osteoarthritis

## Abstract

The cervical spine ligaments, including the anterior longitudinal ligament (ALL) and posterior longitudinal ligament (PLL), play a key role in maintaining spinal stability by limiting excessive movements. This study investigates how ageing affects the mechanical properties of these ligaments. We analysed 33 samples from 12 human cervical spines (15 ALL, 18 PLL), averaging data from the same donors for independent analysis, resulting in 18 final samples (8 ALL, 10 PLL). To explore age-related changes, we classified the samples into two groups—below and above 50 years old—aligning with the peak incidence of major musculoskeletal disorders. The investigation concentrated on the effects of age on four mechanical parameters of the uniaxial stress–stretch curve: initial tangent stiffness ($E_{0}$), maximum tangent stiffness ($E_{m}$), ultimate stress ($P_{u}$) and ultimate stretch ($\lambda_{u}$). When the age effect is neglected, then the behaviours of both the ALL and PLL appeared similar. However, when introducing age as a variable into the context of the ALL and PLL, statistically significant differences became evident. The findings underscored a reduction in maximum tangent stiffness (*p*-value = 0.0147), ultimate stress (*p*-value = 0.0009), and ultimate stretch (*p*-value = 0.0024) when the ALL and PLL were grouped under and above 50 years as a consequence of ageing.

## 1. Introduction

The basic functionalities of cervical spine ligaments are to protect the spinal cord and stabilise the spinal column. They provide stability and contribute to spinal motion patterns [1]. During rear as well as frontal crash accidents, cervical spine ligaments are frequently injured [2,3,4,5,6,7,8]. Low-speed collisions can also alter the functionalities of cervical spine ligaments [9,10,11]. In previous studies, the tensile mechanical properties of the three main human cervical spine ligaments were discussed [12,13,14,15,16,17,18,19]. Most of the studies [12,14,18,20] used samples from older patients (mean age 66 years). In a few studies, age-related changes in isolated spine ligaments (mean age 52.1 years) were investigated [20]. There is increasing interest within both the automotive industry and the military to ascertain the material properties of cervical spinal ligaments because of the morbidity associated with them [21].

The ultimate strength of materials tends to diminish as age increases [22]. Conversely, yield strength and stiffness display augmentation with the progression of age [23]. The process of ageing induces alterations in the diameter of collagen fibres and triggers the relaxation of collagen fibrils that are typically crimped in nature [24,25]. Both the structural attributes of bone–ligament–bone complexes (frequently employed for tensile evaluations of ligaments) and the inherent material properties of ligamentous substances experience a decline due to the ageing process [22,26,27,28,29]. This phenomenon has been observed in studies involving both animal models [22] and human subjects [26,27,28,29].

Most of the mentioned research focuses on individuals over 50 years old. However, Mattucci [30] studied cervical spine ligaments from younger donors (27 to 50 years old) to assess the effects of loading rate, spinal level, and gender at strain rates relevant to automotive crashes. They found that with increasing age, failure force and stiffness decrease. The study also found that higher deformation rates lead to increased stiffness, modulus, failure force, and failure stress, with statistically significant effects observed in the anterior longitudinal ligament (ALL) and posterior longitudinal ligament (PLL).

In the investigation conducted by Neumann [20], parameters such as ultimate load, ultimate stress, stiffness, and energy absorption of the human lumbar anterior longitudinal ligament exhibited a decrease in effectiveness with advancing age. Specimens from younger individuals (34 ± 4 years old) demonstrated more than twice the strength and 50% greater stiffness compared to those obtained from older individuals (77 ± 5 years old). In a study about severe cervical spine injuries conducted by Pintar and Yoganandan [31], it was determined that the tolerance characteristics of the human cervical spine diminish approximately 2.5 times with age. This underscores the importance of accounting for age-related mechanical properties when creating models of tissue responses, in conjunction with considerations for anatomical alterations.

The anatomical composition, spatial arrangement, and operational role of cervical spine ligaments reaffirm the influence of both the age and sex of the donor on the mechanical response of cervical spine ligaments [31,32,33]. These characteristics impact the mechanical properties of tissues, which are relatively more feasible to evaluate compared to the complex effects of illnesses, medications, physical fitness, etc. Nonetheless, there is a paucity of direct investigations analysing the direct effects of ageing on isolated human cervical spine ligaments. Consequently, the available experimental literature lacks sufficient data concerning the influence of age on the mechanical behaviour of cervical spine ligaments.

This presented study, which is a continuation of the previous PhD research conducted in our department by Trajkovski et al. [34,35,36,37], focuses on four important parameters to analyse the effect of age, and they are initial tangent stiffness ($E_{0}$), maximum tangent stiffness ($E_{m}$), ultimate stress ($P_{u}$), and ultimate stretch ($\lambda_{u}$). These parameters are pivotal, as they provide essential insights into the structural behaviour of materials under different conditions, particularly regarding age-related changes.

Firstly, the initial tangent stiffness serves as a foundational measure, reflecting the material’s stiffness at the onset of loading. It indicates the material’s ability to resist deformation initially, offering a baseline for understanding how it responds to stress over time. Secondly, the maximum tangent stiffness represents the peak stiffness achieved by the material during loading. This parameter is significant, as it highlights the maximum capacity of the material to withstand stress before reaching its ultimate limit. Thirdly, the ultimate stress is a critical factor in assessing material strength. It signifies the highest level of stress that the material can endure before failure, providing valuable information on its overall resilience and durability. Lastly, the ultimate stretch indicates the extent to which the material can deform before failure. Understanding this parameter is crucial for predicting the material’s performance and longevity, especially in applications where deformation tolerance is a key consideration.

The decision to limit cadaver specimens to individuals under 50 years old was driven by practical and scientific considerations. Osteoporosis prevalence increases near age 50 [38], influencing ligament properties. The peak age for the onset of MSK disorders and the associated Disability-Adjusted Life Years (DALYs) was between 50 and 54 years [39]. This peak age suggests that the incidence and burden of these disorders are highest in this age range. Recognising the significance of age 50 is crucial for early intervention, prevention, and management strategies in addressing the global burden of MSK disorders. To check the significance of age on the above-mentioned parameters, we have classified our 18 test samples into two categories: the ALL vs. PLL (without considering the age effect) and the ALL and PLL under 50 years old and above 50 years old. While similar research has been conducted on the lumbar spine, our study uniquely explores how age impacts the ligament properties in the cervical spine. To the best of our knowledge, the only prior study examining age-related changes in the ALL is by Neumann [20], and no other studies have comprehensively addressed such changes in cervical spinal ligaments. When the age effect is neglected, we do not observe significant differences between the ALL and PLL. However, when we classify the ALL and PLL together, under and above 50 years old, statistical differences begin to emerge.

## 2. Materials and Methods

**Figure 1:**
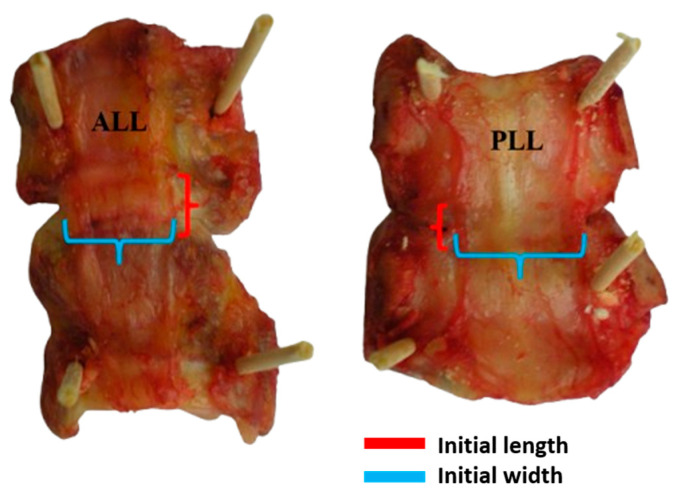
Isolated bone–ligament–bone specimens of the ALL and PLL samples with their initial length (red) and initial width (sky blue).

The use of human cadaveric tissues in this study was approved by the Commission for Medical Ethics, Ministry of Health, Republic of Slovenia, approval code: 690/00. All procedures were conducted in accordance with ethical guidelines. Informed consent for the donation of cadaveric tissues was obtained in compliance with legal and ethical requirements. Sample preparation and uniaxial testing methods are well described in the previous work by Trajkovski et al. [34,35,36,37]. Samples from 12 isolated human cervical spines were tested in tension up to failure. The cadaver age ranged from 23 to 84 years, with an average height of 1761.70 mm and weight of 85.33 kg. Fresh cervical spine samples (24–48 h after donor death) and sample preparation were provided by the Institute of Forensic Medicine and the Institute of Anatomy at the Faculty of Medicine, University of Ljubljana. Subsequent to the isolation process, the dimensions of the ligament were assessed using a conventional contact method, employing digital callipers for measurement. The width and thickness of the ligament were measured at the midpoint and between the adjacent vertebrae, using digital callipers as shown in Figure 1. These measurements were employed as the major and minor axes of an ellipse, effectively approximating the ligament’s cross-sectional area (CSA). It is worth acknowledging that the ligament’s width and thickness exhibit variation along the spinal column. Nevertheless, for the sake of simplification, the CSA was assumed to remain constant along the length of the ligament, with measurements taken at the midpoint considered the smallest CSA.

**Figure 2:**
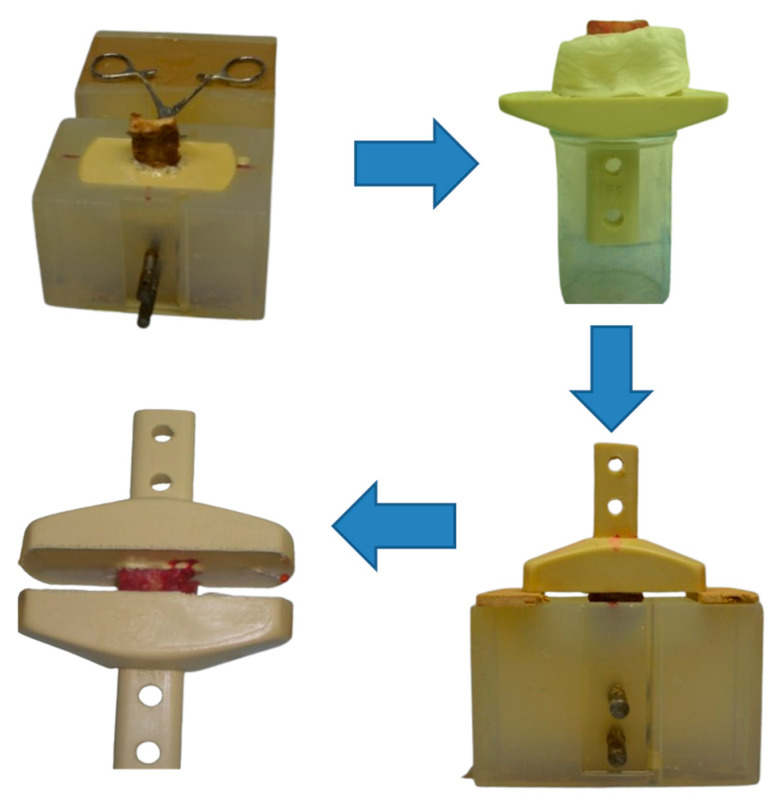
Scheme of specimen-gripping preparation for lower and upper vertebrae.

After defining the dimensions of the samples, the bony part of the ALL and PLL sample was impregnated with a two-component polyurethane adhesive that quickly hardens in the shape of the desired casting and forms a gripper that allows the specimens to be attached to the test device, as shown in Figure 2.

**Figure 3:**
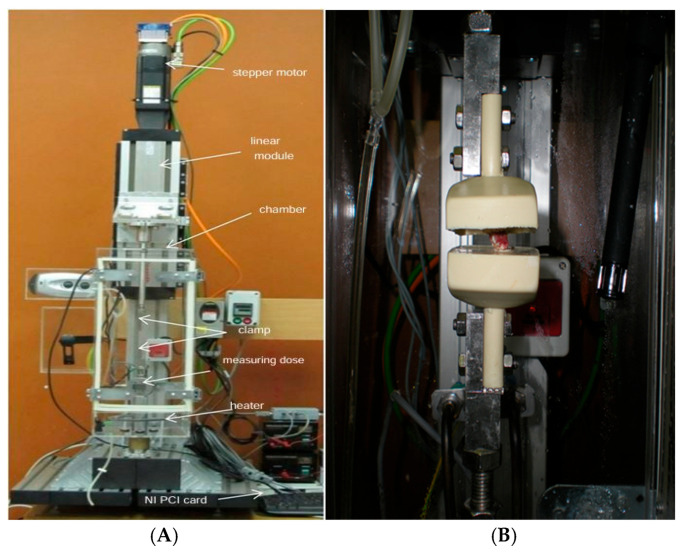
(**A**) Custom designed Testing machine and (**B**) testing clamps with specimen.

Uniaxial tension tests were conducted in conditions mimicking physiological settings. These conditions were carefully controlled, with the air temperature maintained at 36.6 ± 0.5 °C and the air humidity at 94 ± 0.5%. The tests were conducted using a custom-designed mechanical testing apparatus, as illustrated in Figure 3A. Figure 3B highlights the testing clamps along with the specimen. The core component of this apparatus was a stepper motor (specifically, model VRDM 3913 from Berger Lahr, Ljubljana, Slovenia) integrated into a linear compact module featuring a threaded spindle (model CKK 20-145 from Bosch Rexroth, Ljubljana, Slovenia). The entire system was computer-controlled via a Stepper Drive controller (model SD326 from Berger Lahr, Ljubljana, Slovenia).

All experiments were performed within a thermostatic chamber designed to replicate physiological conditions. This chamber was constructed from plexiglass and had dimensions of 200 × 200 × 596 mm, allowing for the observation of the sample throughout testing. To regulate the temperature and humidity, the lower section of the chamber was filled with water, which was maintained at approximately 38 ± 8 °C. This water layer contributed to elevating the humidity of the air above it within the chamber.

The specimens were ramped at a slow displacement rate of 0.05 mm/s to 5 N and allowed to relax for 1000 s. The resulting displacement was recorded and used as the reference ligament length for the calculation of the engineering strain. After the specimen was fully relaxed, it was subjected to a constant displacement rate of 2 mm/s until failure. Data on applied displacement and measured force were recorded with 10 Ks/s. From these experiments, force–displacement curves and maximal force and displacement at failure were determined. The force at which further elongation does not result in further force increase was defined as failure load. The accompanying elongation was defined as failure elongation. Linear stiffness was calculated as the slope of the linear part of the nominal stress–stretch curve. Based on load elongation data (*F-*∆l), initial cross-sectional area ($A_{0}$), and initial length ($l_{0}$), stress–stretch curves (*P-λ*) were created. First Piola Stress and the stretch ratio (λ) were calculated as follows:

The stress *P* is given by the force per unit initial cross-sectional area.

(1) $$P=\frac{F}{A_{0}}$$

Strain (ε) is defined as the change in length (Δl) divided by the original length ($l_{0}$).

(2) $$\varepsilon =\frac{\Delta l}{l_{0}}$$

Using this, the stretch ratio (λ) was calculated as follows:

(3) $$\lambda =1+\varepsilon =1+\frac{\Delta l}{l_{0}}$$

**Figure 4:**
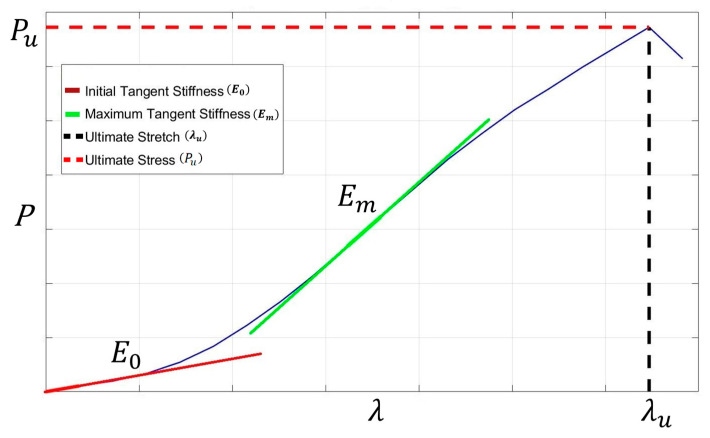
Different markers to analyse the age effect.

Once we obtained the stress vs. stretch curves, we focused on understanding the effect of age on four mechanical markers of ligaments, namely: (i) initial tangent stiffness, $E_{0}$; (ii) maximum tangent stiffness, $E_{m}$; (iii) ultimate stress, $P_{u}$; and (iv) ultimate stretch, $\lambda_{u}$. These can be seen in Figure 4.

To remove the high-frequency noise, the data were filtered using a low-pass filter into MATLAB R2021a (academic use). Initial tangent stiffness was calculated as the slope of a linear fit of the stress–strain curve up to a 10% stretch. The maximum tangent stiffness was calculated as the maximum value of the tangent stiffness, which is the change in stress divided by the change in strain between consecutive points. The MATLAB script finds this maximum value of the slope and its corresponding index.

Statistical analysis: We evaluated the data for homogeneity of variances and normality to ensure appropriate statistical testing. In this study, the Lilliefors test was used to evaluate whether the data followed a normal distribution. This test compares the empirical data distribution to a theoretical normal distribution, generating two outputs: a binary test decision (h) and a *p*-value (*p*). A value of h = 1 indicates that the data do not follow a normal distribution, while h = 0 suggests normality. The result shows that all data come from a normal distribution. Statistical difference was calculated using MATLAB with a two-sample *t*-test, which checks if there is a significant difference between the means of two groups. The code computes the mean, median, and standard deviation for each group. The ttest2 function then compares the two groups and returns a *p*-value. If the *p*-value is less than a set threshold (usually 0.05), it indicates that the difference between the groups is statistically significant.

The following cases are considered for analysing ligament types and the effect of age:

1. Effect of ligament type only, i.e., the ALL vs. PLL without considering the age effect.
2. Effect of age only, i.e., the ALL+PLL under 50 years vs. the ALL+PLL above 50 years, without considering the ligament type.

## 3. Results

**Figure 5:**
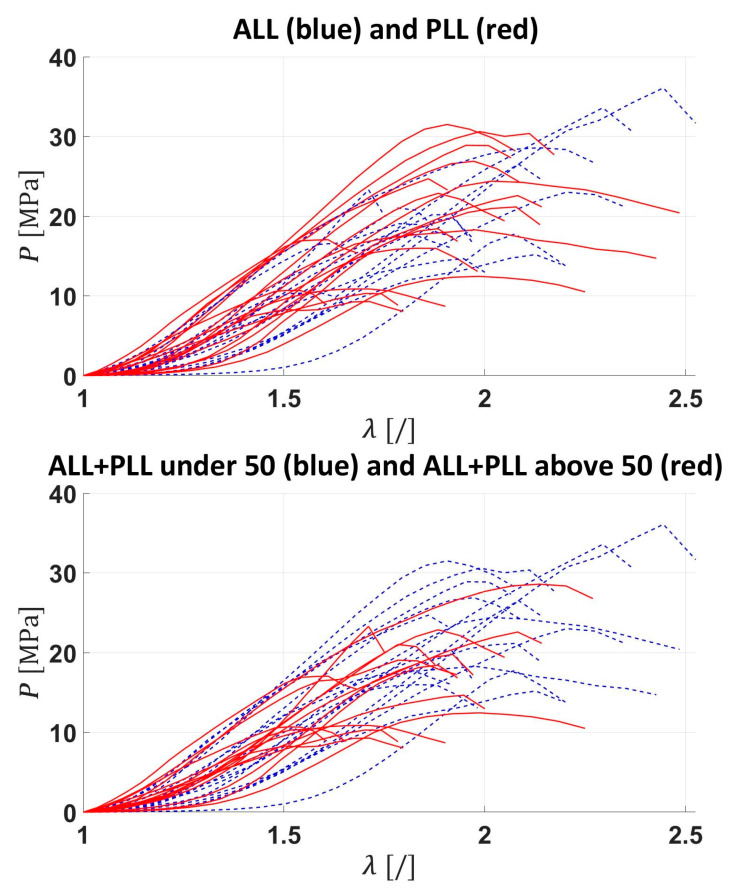
Stress vs. stretch curves for the ALL vs. PLL and ALL+PLL under and above 50.

The stress–stretch curves for the ligaments are shown in Figure 5, grouped by family of ligaments and by age group. Independent of age and ligament type, the stress–stretch curve shows the J-type response observed in fibrous soft tissue with an initial compliant response, followed by gradual stiffening until reaching a maximum value, where the ligament’s stress–stretch curve becomes linear until tissue damage starts, characterised by a gradual reduction in tangent stiffness until reaching a maximum. In most cases, the failing mode corresponded with a gradual delamination of the tissue, leading to rather smooth stress softening.

Table 1 summarises the investigated mechanical markers for the two ligament types in both age ranges considered in this study. For both ligament types, the results show a lower average value of the ultimate stress and ultimate stretch above 50 years.

### 3.1. Effect of Ligament Type Only

**Figure 6:**
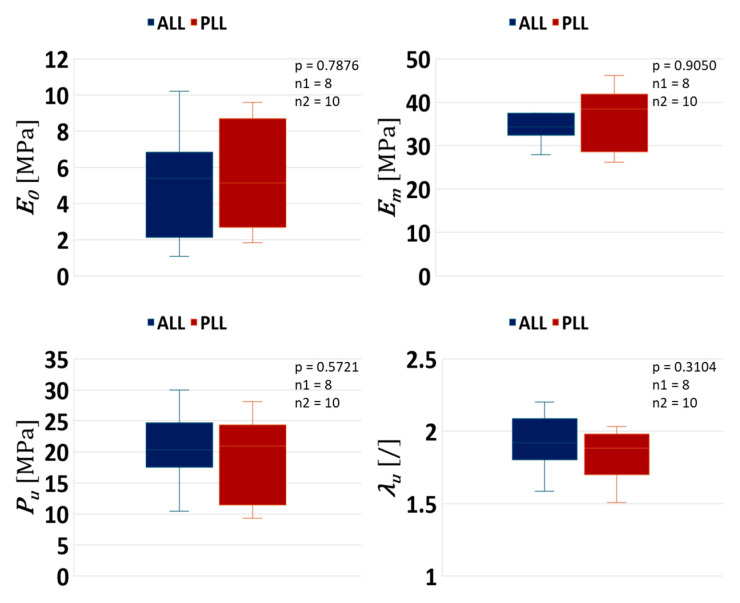
Statistical difference between the ALL vs. PLL. Statistical difference is indicated by an asterisk, and n1 and n2 represent the sample size of the ALL and PLL, respectively.

Figure 6 shows a comparison between the ALL and PLL without considering the effect of age.

From Figure 6, it can be seen that there are no statistical differences in any of the four mechanical markers of the ALL and PLL if the age effect is neglected.

### 3.2. Effect of Age Only

**Figure 7:**
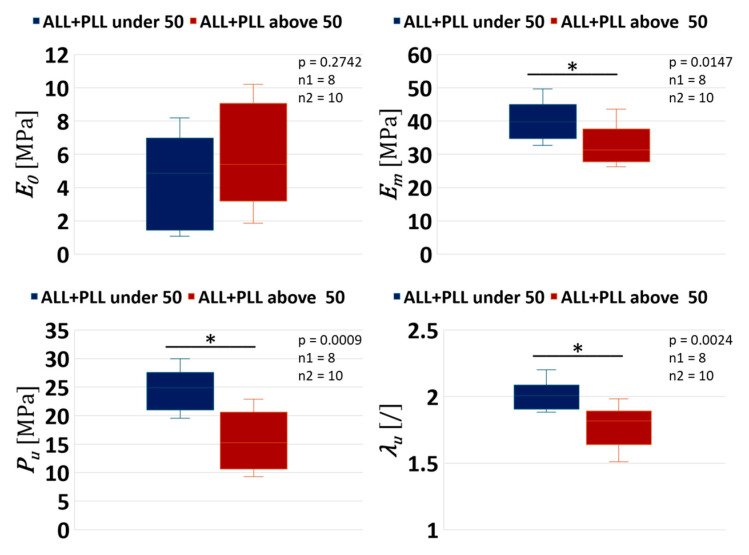
Statistical difference between the ALL+PLL under 50 years vs. the ALL+PLL above 50 years. Statistical difference is indicated by an asterisk, and n1 and n2 represent the sample size of the ALL+PLL under 50 years and the ALL+PLL above 50, respectively.

The ALL+PLL under 50 years vs. the ALL+PLL above 50 years, without considering the ligament type, was used in this analysis. Figure 7 shows the results for the range of age, independent of ligament type.

Figure 7 shows significant differences in $E_{m}$ (*p*-value = 0.0147), $P_{u}$ (*p*-value = 0.0009), and $\lambda_{u}$ (*p*-value = 0.0024), with a larger value of maximum tangent stiffness, ultimate stress, and ultimate stretch for ligaments under 50 years. On the contrary, no significant difference was found for $E_{0}$ (*p*-value = 0.2742).

These findings underscore the impact of age on mechanical properties within ligaments, shedding light on age-related alterations in ligament behaviour.

## 4. Discussion

In this presented study, the biomechanical behaviour of the two main cervical ligaments (ALL and PLL) obtained from the human cadaveric cervical spines was analysed. We determined the influence of donor age on the mechanical response of ligaments being characterised by four well-defined parameters, two related to the stiffness of the ligaments, i.e., the initial and the maximum tangent stiffness, and two related to the resistance to failure, i.e., ultimate stress and stretch. This study does not aim to address questions beyond a simple uniaxial test. Instead, it focuses on analysing specific mechanical parameters under uniaxial loading to investigate age-related changes in ligament properties.

The results indicated no significant differences in any of these parameters between the ALL and PLL when the age effect is not considered. Both of them are longitudinal ligaments, which share a comparable histological composition, anatomical location concerning the spinal cord, and function, and they exhibit a congruent mechanical response [40,41]. It is important to note that, on average, the PLL demonstrates greater stiffness than the ALL [30]. In other studies, it has been reported that the ultimate load and ultimate stress of the PLL tend to be higher than those of the ALL, but no significant difference was observed [17,21]. We also do not see any statistical difference between the ALL and PLL when the age effect is neglected.

If the age difference is considered independent of ligament type, i.e., the ALL+PLL under 50 years vs. the ALL+PLL above 50 years, some interesting differences emerge. In particular, we observed a significant reduction in the maximum tangent stiffness, ultimate stress, and ultimate stretch of ligaments as age increased, which can be seen in Figure 7. This aligns well with the previous studies as follows: Neumann [20], involving 15 human lumbar anterior longitudinal ligaments sourced from individuals spanning an age range of 30 to 80 years were studied. In this study, significant age-related declines in the ultimate load, ultimate stress, stiffness, and energy absorption of the human lumbar anterior longitudinal ligament are reported. Ageing results in the disappearance of oxytalan fibres, which play a crucial role in providing tissue resistance [42]. Furthermore, another study revealed noteworthy findings regarding the relationship between age and various mechanical properties of the ligaments. Specifically, a significant negative correlation was observed between age and ultimate stress, as well as between age and stiffness [43]. Another study used 30 samples and found significant differences in the ultimate stress of the patellar tendon complex between younger (29–50 years) and older (64–93 years) age groups [44]. Another study on porcine knees found a significant negative correlation between age and both ultimate stress and ultimate stretch [45]. Artificial intelligence and machine learning tools can further help to understand these changes [46,47]

Given that five major musculoskeletal (MSK) disorders—low back pain, neck pain, osteoarthritis, rheumatoid arthritis, and gout—tend to peak between the ages of 50 and 54, we have categorised our data into two distinct age groups: below and above 50 years old [39]. This classification allows us to analyse the effects of ageing on ligament properties more clearly, treating age as a discrete variable.

Discretizing age in this way can be crucial for identifying specific trends or differences that might be obscured when age is treated as a continuous variable. By focusing on these two age groups, we can better understand how the mechanical properties of ligaments, such as $P_{u}$ and $\lambda_{u}$, change with age, particularly with the onset of MSK disorders.

The observed decrease in ultimate stress, ultimate stretch, and maximum tangent stiffness with age may be associated with the structural and functional changes in the ligaments as they weaken or become less elastic over time. These changes could contribute to the increased susceptibility to MSK disorders, as the ligaments may no longer provide adequate support or flexibility, leading to joint instability, reduced range of motion, and greater vulnerability to injury or chronic conditions like those observed in the major MSK disorders. By analysing these variables with age, we gain insights into the biomechanical factors that may underlie the prevalence of these disorders in individuals over 50. This explanation ties the importance of age discretization to the specific research goals and potential biomechanical implications, providing a clear rationale for the approach. Very recent research [48] indicates that ageing does not occur at a constant rate throughout life. Instead, there are specific periods around the ages of 44 and 60 years where the rate of ageing appears to accelerate. Ageing impacts the extracellular matrix (ECM), which plays a crucial role in tissue structure and function. This study found that the composition and regulation of the ECM change significantly with age, particularly during the transitions at 44 and 60 years. This could lead to alterations in the structural stability, mechanical strength, elasticity, and hydration of tissues and cells.

The collected stress–strain curves and data for the ALL and PLL provide key parameters such as ultimate stress, ultimate stretch, maximum tangent stiffness, and initial tangent stiffness. These data gathered across the overall population, and specifically for individuals under and over 50 years of age, can be effectively utilised for computational modelling, including Finite Element Method (FEM) simulations. The availability of data for individuals under and over 50 years old allows for the inclusion of age-specific variations in computational models. This enhances the accuracy of simulations by reflecting how ligament properties may change with age, which is important for predicting the risk of musculoskeletal (MSK) disorders, such as low back pain, neck pain, osteoarthritis, rheumatoid arthritis, and gout, or evaluating treatment options in different age groups. These data are valuable for refining computational models, ensuring they account for variations in ligament behaviour due to ageing or other factors. This study also highlights the increased vulnerability of ligaments in individuals over 50, suggesting the need for age-specific injury prevention strategies, such as targeted exercises to strengthen ligaments and improve flexibility. In surgical interventions, adaptations to account for weaker ligaments may be necessary, using additional fixation or supportive materials. For rehabilitation, focusing on restoring ligament strength and stability can enhance recovery and reduce reinjury risk in older patients. A recent study also examines the application of mechanical principles in medical treatments, with a focus on bone mechanobiology and cancerogenesis. It highlights the use of computational models and in silico trials to create personalised therapies [49]

The study presented here has certain limitations, which are well described in previous research by Trajkovski et al. [34,35,36,37], that need to be considered when interpreting the results. A limitation of our study is its small sample size, which may reduce the reliability of the findings and limit their applicability to the broader population, as it may not capture enough variation to reflect true trends. Due to the limited number of specimens, we were unable to analyse the effects of gender, activity level, and health conditions on ligament properties, which would have provided a more comprehensive understanding. The deformation was measured on a machine crosshead under the assumption that deformation in the ligament-to-bone attachment zone could be neglected, especially compared to ligament deformation in the middle, between adjacent vertebras. The ligament’s width and thickness were measured at the midpoint using digital callipers and approximated as an ellipse’s major and minor axes to calculate the cross-sectional area (CSA). We acknowledge that while this method provides practical approximation, advanced techniques like Digital Image Correlation (DIC) or Digital Volume Correlation (DVC) [50] would offer more accurate predictions of large deformations. Such methods could capture the complex deformation patterns and variability in ligament dimensions more effectively, providing deeper insights into their biomechanical behaviour. During the experiments, the same protocol was applied to all samples, ensuring minimised experimental uncertainty. However, biological uncertainties are currently joined and therefore limited to the factor of age, and our future study will address this issue by microscopic analysis of different unloaded and loaded samples, as mentioned before. The measured mechanical properties in our study are comparable to the mechanical properties in the Mattucci study [30]. Also, similar physiological conditions were used by [30,51]. However, we used fresh samples, but Mattucci used frozen samples, and that could be a reason for the difference in stiffness.

## 5. Conclusions

This study demonstrates significant age-related changes in the mechanical properties of cervical spine ligaments. Specifically, the anterior and posterior longitudinal ligaments exhibit reductions in maximum tangent stiffness, ultimate stress, and ultimate stretch in individuals over 50 compared to those under 50. When age was not accounted for, the mechanical responses of both ligaments appeared similar. However, introducing age as a variable revealed distinct differences, highlighting the importance of considering age both as a discrete and continuous variable to fully understand its impact on ligament behaviour.

## Figures and Tables

**Table 1:** Material properties of cervical spine ligaments, mean values ± standard deviation.

Properties	ALL	PLL	*p*-Values	Sample Size	ALL+PLL Under 50	ALL+PLL Over 50	*p*-Values	Sample Size
Initial tangent stiffness (MPa)	5.01 ± 3.76	5.52 ± 4.52	0.7876	ALL—8, PLL—10	4.49 ± 2.87	6.08 ± 3.04	0.2742	ALL+PLL under 50—8, ALL+PLL over 50—10
Maximum tangent stiffness (MPa)	35.93 ± 8.50	37.23 ± 8.37	0.9050	ALL—8, PLL—10	40.08 ± 5.77	32.70 ± 5.63	0.0147	ALL+PLL under 50—8, ALL+PLL over 50—10
Ultimate stress (MPa)	21.80 ± 7.00	19.83 ± 7.25	0.5721	ALL—8, PLL—10	24.44 ± 3.68	15.68 ± 5.11	0.0009	ALL+PLL under 50—8, ALL+PLL over 50—10
Ultimate stretch (-)	1.99 ± 0.24	1.87 ± 0.16	0.3104	ALL—8, PLL—10	2.01 ± 0.11	1.77 ± 0.15	0.0024	ALL+PLL under 50—8, ALL+PLL over 50—10

## Data Availability

Available upon request.
